# National Diabetes Month — November 2013

**Published:** 2013-11-01

November is National Diabetes Month. In 2010, approximately 26 million persons in the United States had diabetes, and an estimated 79 million adults had prediabetes (1). Testing for diabetes is recommended for adults with certain risk factors, including being aged ≥45 years, being overweight or obese, having a family history of diabetes or a history of gestational diabetes, and being physically inactive (2). Persons with diabetes can take steps to control the disease and prevent complications, and those with prediabetes can prevent or delay the onset of type 2 diabetes through weight loss and physical activity (1,3).

CDC and state and territorial public health programs, in collaboration with other partners, work to improve outcomes for persons with diabetes and to reduce the incidence of type 2 diabetes. For example, CDC’s National Diabetes Prevention Program (http://www.cdc.gov/diabetes/prevention) supports the nationwide implementation of community-based lifestyle change programs for persons at high risk for type 2 diabetes. CDC’s Native Diabetes Wellness Program (http://www.cdc.gov/diabetes/projects/diabetes-wellness.htm) assists 17 American Indian and Alaska Native communities in increasing access to traditional local foods and participation in physical activity. The program’s series of *Eagle Books* for children aged 4–13 years teach respect for traditional ways of health, including drinking water, eating local foods, and being active. In addition, the National Diabetes Education Program (http://www.yourdiabetesinfo.org), jointly sponsored by CDC and the National Institutes of Health, provides tools and resources to help organizations and individuals address diabetes in their communities, health-care practices, and businesses.
